# Pan-cancer analysis reveals potential of FAM110A as a prognostic and immunological biomarker in human cancer

**DOI:** 10.3389/fimmu.2023.1058627

**Published:** 2023-02-27

**Authors:** Hongguang Zhong, Qianqian Shi, Qin Wen, Jingyi Chen, Xuan Li, Ruiwen Ruan, Shaocheng Zeng, Xiaofeng Dai, Jianping Xiong, Li Li, Wan Lei, Jun Deng

**Affiliations:** ^1^ Department of Oncology, The First Affiliated Hospital of Nanchang University, Nanchang, Jiangxi, China; ^2^ Jiangxi Key Laboratory for Individual Cancer Therapy, Nanchang, Jiangxi, China; ^3^ Department of Pathology, The First Affiliated Hospital of Nanchang University, Nanchang, Jiangxi, China

**Keywords:** FAM110A, pan-cancer analysis, bioinformatics, prognosis, immune infiltration

## Abstract

**Background:**

Despite great success, immunotherapy still faces many challenges in practical applications. It was previously found that family with sequence similarity 110 member A (FAM110A) participate in the regulation of the cell cycle and plays an oncogenic role in pancreatic cancer. However, the prognostic value of FAM110A in pan-cancer and its involvement in immune response remain unclear.

**Methods:**

The Human Protein Atlas (HPA) database was used to detect the expression of FAM110A in human normal tissues, the Tumor Immune Estimation Resource (TIMER) and TIMER 2.0 databases were used to explore the association of FAM110A expression with immune checkpoint genes and immune infiltration, and the Gene Set Cancer Analysis (GSCA) database was used to explore the correlation between FAM110A expression and copy number variations (CNV) and methylation. The LinkedOmics database was used for Gene Ontology (GO) and Kyoto Encyclopedia of Genes and Genomes (KEGG) pathway enrichment analysis. Statistical analysis and visualization of data from the The Cancer Genome Atlas (TCGA) or the Genotype–Tissue Expression (GTEx) databases were performed using the R software (version 3.6.3). Clinical samples were validated using immunohistochemistry.

**Results:**

FAM110A expression was elevated in most tumor tissues compared with that in normal tissues. CNV and methylation were associated with abnormal FAM110A mRNA expression in tumor tissues. FAM110A affected prognosis and was associated with the expression of multiple immune checkpoint genes and abundance of tumor-infiltrating immune cells across multiple types of cancer, especially in liver hepatocellular carcinoma (LIHC). FAM110A-related genes were involved in multiple immune-related processes in LIHC.

**Conclusion:**

FAM110A participates in regulating the immune infiltration and affecting the prognosis of patients in multiple cancers, especially in LIHC. FAM110A may serve as a prognostic and immunological biomarker for human cancer.

## 1 Introduction

With the successful application of several immune checkpoint blockers (ICBs), including PD-1, PD-L1, and LAG-3 antagonists, immunotherapy is now a powerful and critical treatment approach (1). However, immunotherapy responders account for only a small fraction of patients with cancer, and resistance to immunotherapy exists in the treatment of most tumor types and patients with cancer (2–4). The tumor microenvironment (TME), which plays a critical role in tumorigenesis and tumor progression, is an important factor influencing the efficacy of immunotherapy (5), and therapeutic strategies targeting the TME have also been regarded as a novel promising modality for the treatment of cancers in recent years (6). However, the complex mechanisms involved in regulating the formation and dynamic variation of TME remain unclear. As a result, the identification of novel prognosis and TME-related genes will help overcome the bottlenecks that immunotherapy is currently facing.

The family with sequence similarity 110 (FAM110), which includes three members, FAM110A, FAM110B, and FAM110C, has been demonstrated to be centrosome-related. They are located in centrosomes and accumulate at spindle poles during mitosis (7). Increasing studies have indicated that FAM110 family protein participates in carcinogenesis. FAM110A exerts an oncogenic role by facilitating malignant biological behaviors of pancreatic cancer cells (8). FAM110B modulates the biologic behavior by inhibiting Wnt/β-catenin signaling in non-small cell lung cancer (9) and has been identified as a potential growth promoting key gene for castration-resistant prostate cancer (10). FAM110C is involved in cell spreading, migration, and filopodia induction (11). Overall, these findings suggest that FAM110 family genes are closely related to malignancies.

Recent studies have revealed that FAM110A expression is regulated by the cell cycle and is highly expressed in the G2 phase; Depletion of FAM110A leads to mitotic defects and delays mitotic progression (12). In lymphoid tissues, proliferation signals from antigen-presenting cells simulated by Dynabeads CD3/CD28 can significantly activate FAM110A expression in CD4+ T lymphocytes (7). These findings indicate a potential role for FAM110A in promoting tumor cell proliferation and immune cell infiltration. However, research on FAM110A, particularly regarding the relevance of immune responses in cancer, is currently inadequate. A more comprehensive analysis of FAM110A is warranted to better understand its functional roles in malignancies.

In this study, we employed a series of bioinformatics approaches to conduct pan-cancer analysis of FAM110A from multiple aspects, including gene expression and genomic alterations, correlation with prognosis, immunological markers, immune infiltration, and gene sets of interest. Moreover, immunohistochemical (IHC) analyses were performed to further confirm the role of FAM110A in LIHC. Our results revealed that FAM110A expression is correlated with immune response and may be a promising prognostic biomarker in multiple cancers.

## 2 Results

### 2.1 FAM110A expression in various human normal tissues

**Figure 1:**
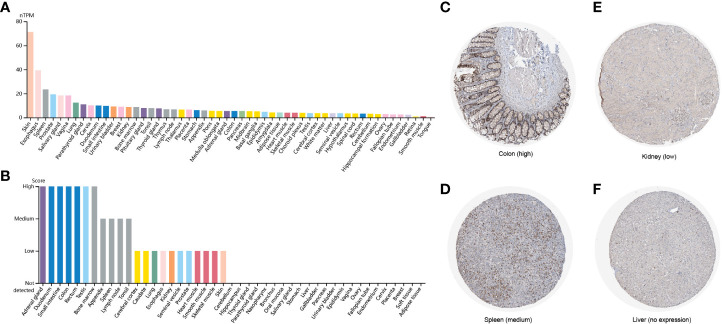
FAM110A expression in various human normal tissues. **(A)** FAM110A mRNA expression profiles in normal human tissues. **(B)** FAM110A protein expression data in human normal tissues. **(C–F)** Representative IHC images of FAM110A expression in normal colon, spleen, kidney, and liver tissues.

To explore the expression levels of FAM110A in various types of normal human tissues, we evaluated the mRNA and protein expression of FAM110A using the Human Protein Atlas (HPA) database. As shown in **Figure 1A**, the tissues with the highest FAM110A expression were the skin, esophagus, spleen, prostate, and vagina. Next, we examined its expression at the protein level. We found that the expression levels of FAM110A in various tissues were significantly different (**Figure 1B**). FAM110A mRNA and protein showd different expression patterns in normal tissues, this may be due to the low specificity of the FAM110A antibody, which has not been experimentally validated. Immunohistochemistry showed that FAM110A was expressed in the nucleus and cytoplasm, and representative tissue staining results for different expression levels were shown **(** **Figures 1C–F**), including colon (high), spleen (medium), kidney (low), and liver (no expression).

### 2.2 FAM110A expression in various tumor tissues

**Figure 2:**
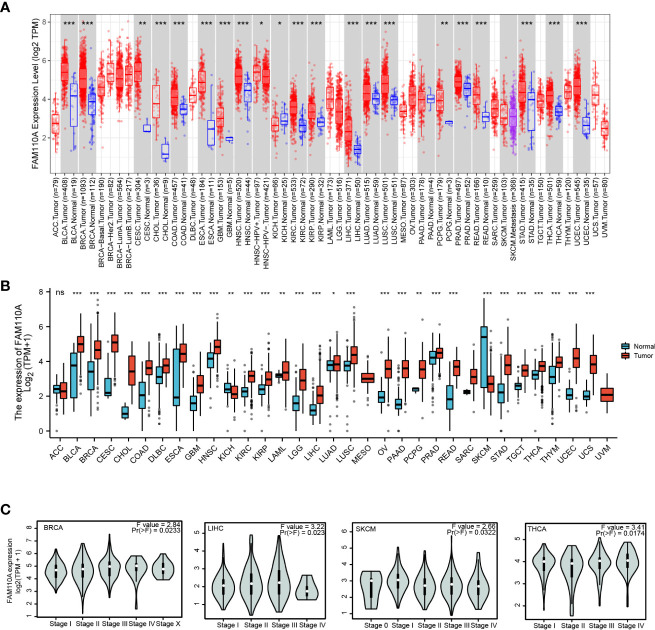
FAM110A expression in various tumor tissues. **(A)** FAM110A expression levels in pan-cancer from TCGA database were analyzed by TIMER2.0. (*P<0.05, **P<0.01, ***P<0.001). **(B)** FAM110A expression differences between tumor and normal tissues in pan-cancer from the TCGA and GTEx databases. (*P<0.05, **P<0.01, ***P<0.001). **(C)** The correction between FAM110A expression and the pathological stages of BRCA, LIHC, SKCM and THCA using the GEPIA2 database.

We employed TIMER 2.0 website to explore the expression changes of FAM110A between tumor tissues and correspond normal tissues in the TCGA database. As shown in **Figure 2A**, the mRNA expression of FAM110A was significantly increased in bladder urothelial carcinoma (BLCA), breast invasive carcinoma (BRCA), cervical squamous cell carcinoma (CESC), cholangiocarcinoma (CHOL), colon adenocarcinoma (COAD), esophageal carcinoma (ESCA), glioblastoma multiforme (GBM), head and neck squamous cell carcinoma (HNSC), kidney renal clear cell carcinoma (KIRC), kidney renal papillary cell carcinoma (KIRP), LIHC, lung adenocarcinoma (LUAD), lung squamous cell carcinoma (LUSC), pheochromocytoma and paraganglioma (PCPG), prostate adenocarcinoma (PRAD), rectum adenocarcinoma (READ), stomach adenocarcinoma (STAD), thyroid carcinoma (THCA), uterine corpus endometrial carcinoma (UCEC) and reduced only in kidney chromophobe (KICH).

Due to the small quantity of corresponding normal tissue expression data in the TCGA database, we further conducted a joint analysis with matched normal tissue expression data from the Genotype-Tissue Expression (GTEx) database in a more convincing manner. The expression of FAM110A was elevated in most cancers, including BLCA, BRCA, CESC, CHOL, COAD, diffuse large B-cell lymphoma (DLBC), ESCA, GBM, HNSC, KIRC, KIRP, acute myeloid leukemia (LAML), lower grade glioma (LGG), LIHC, LUAD, LUSC, ovarian serous cystadenocarcinoma (OV), pancreatic adenocarcinoma (PAAD), PCPG, PRAD, READ, STAD, testicular germ cell tumors (TGCT), THCA, thymoma (THYM), UCEC, and uterine carcinosarcoma. In contrast, FAM110A expression in the tumor tissues of KICH and skin cutaneous melanoma (SKCM) was significantly decreased (**Figure 2B**). In addition, we further explored FAM110A expression across different cancer pathological stages using the GEPIA database and found that FAM110A mRNA expression was correlated with clinicopathological stages in BRCA, LIHC, SKCM, and THCA (**Figure 2C**).

### 2.3 Copy number variation and methylation contribute to driving the abnormal expression of FAM110A in pan-cancers

**Figure 3:**
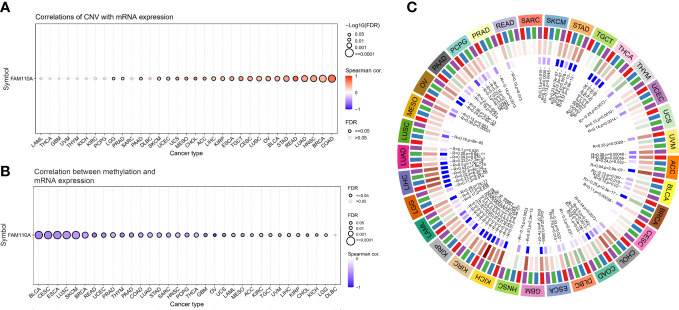
CNA and methylation contribute to driving the abnormal expression of FAM110A in pan-cancers. **(A)** Correlation of CNV and FAM110A mRNA expression in the GSCA database. A significant positive correlation was observed in patients with COAD, BRCA, HNSC and LUAD. **(B)** In most tumor types except DLBC, FAM110A mRNA expression was significantly associated with methylation levels, the strongest correlation was observed in BLCA, CESC, ESCA, LUSC, and SKCM. **(C)** Correlation of FAM110A mRNA with four methyltransferases, namely DNMT1 (Red), DNMT2 (Blue), DNMT3A (Green) and DNMT3B (Purple).

To further explore the mechanisms underlying the abnormal expression of FAM110A mRNA, we analyzed the relationship between gene copy number variation (CNV) and mRNA expression. The results from the GSCA database showed that there was a significant positive correlation between the expression of FAM110A and CNV in patients with COAD, BRCA, HNSC, and LUAD; in contrast, the correlations were not significant in patients with LAML, THCA, GBM, uveal melanoma (UVM), THYM, KICH, KIRC, PCPG, sarcoma (SARC), and DLBC (**Figure 3A**), suggesting that CNV may not be the only factor responsible for abnormal FAM110A expression, and the underlying mechanisms leading to aberrant expression may be inconsistent in different tumors.

DNA methylation is an epigenetic process that can significantly modulate gene transcription (13); therefore, we found that DNA methylation levels were significantly correlated with mRNA expression in most tumor types, especially in BLCA, CESC, ESCA, LUSC, and SKCM (**Figure 3B**). To further explore the mechanisms responsible for the discordance in methylation levels in various cancers, we assessed the correlation between FAM110A and four methyltransferase genes, named DNA methyltransferase 1 (DNMT1), DNMT2, DNMT3A, and DNMT3B, and found a significant correlation between them and FAM110A in STAD, KICH, KIRC, KIRP, and LIHC (**Figure 3C**).

### 2.4 FAM110A expression level correlates with prognosis in cancers

**Figure 4:**
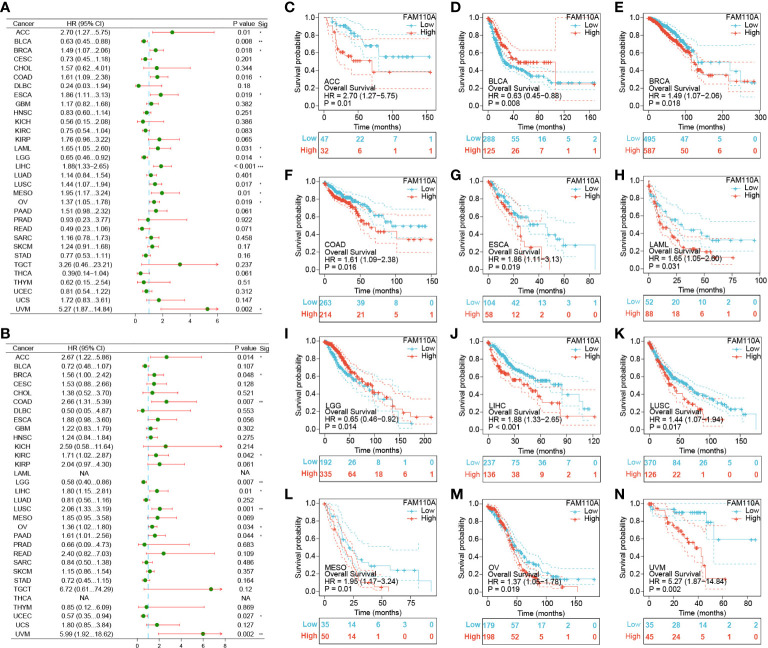
Survival analysis of FAM110A in different types of cancer in the TCGA database. **(A, B)** Correlation of FAM110 expression with OS and DSS in patients with different cancers (*P<0.05, **P<0.01, ***P<0.001). **(C-N)** Kaplan–Meier (KM) curves of OS with significance in 12 cancer types (ACC, BLCA, BRCA, COAD, ESCA, KIRC, LAML, LIHC, LUSC, MESO, OV and UVM) in TCGA.

To further elucidate the effect of FAM110A expression on the prognosis of patients with cancer, we downloaded TCGA RNA-seq and clinical data. Univariate COX regression analysis was performed to explore the relationship between FAM110A expression and overall survival (OS) in 33 cancer types, as shown in **Figure 4A**. High expression of FAM110 was significantly associated with poorer prognosis in patients with adrenocortical carcinoma (ACC), BLCA, BRCA, COAD, ESCA, KIRC, LAML, LIHC, LUSC, mesothelioma (MESO), OV, and UVM, with LIHC showing the most significant association with FAM110A. In contrast, high CDCA4 expression levels were positively associated with better prognosis in BLCA and LGG (**Figures 4C–N**).

To exclude the bias caused by non-tumor events, we further evaluated the effect of FAM110A expression levels on disease-specific survival (DSS) (**Figure 4B**). The results were roughly consistent with the OS analysis, demonstrating that high FAM110A expression was associated with poor prognosis in patients with ACC, BRCA, COAD, KIRC, LIHC, LUSC, MESO, OV, PAAD and UVM, while negatively correlated with prognosis in patients with LGG and UCEC (**Supplementary Figure 1**). These results revealed that FAM110A expression levels are significantly associated with prognosis in patients with multiple tumor types.

### 2.5 Correlation of FAM110A expression on immune checkpoints and immunotherapy

**Figure 5:**
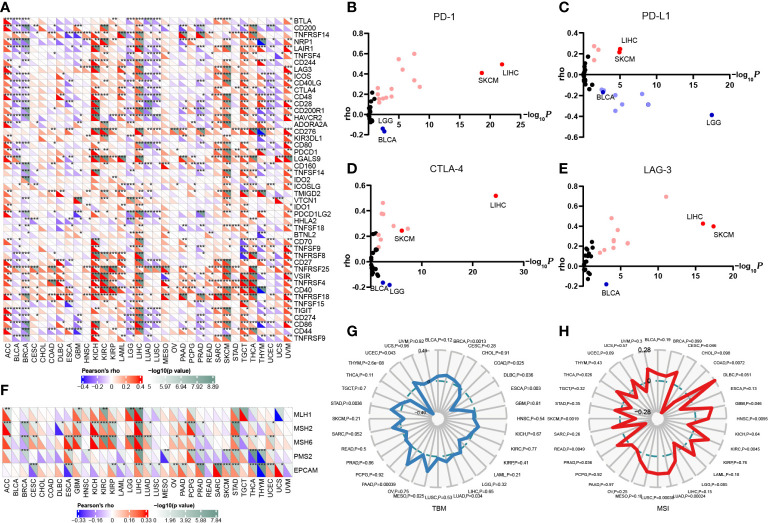
Correlation of FAM110A expression on immune checkpoints and immunotherapy. **(A)** The correlation of FAM110A with more than 40 immune checkpoint genes in pan-cancer (*P<0.05, **P<0.01, ***P<0.001). **(B-E)** The correlation of FAM110A with PD-1, PD-L1, CTLA-4 and LAG-3 in the TIMER 2.0 database. **(F)** The correlation of FAM110A with MMR-relate genes, including MLH1, MLH2, MLH6, PMS2 and EPCAM in pan-cancers (*P<0.05, **P<0.01, ***P<0.001). **(G, H)** The correlations of FAM110A expression and TMB, MSI in pan-cancers.

Since the expression of immune checkpoint genes is closely related to the efficacy of immunotherapy, we first explored the relevance of FAM110A to genes that are recognized as immune response-related checkpoints using the TCGA database. Interestingly, two significant but diametrically opposite trends were observed among the different cancers. FAM110A expression displayed a strong positive correlation with these genes including neuropilin 1 (NRP1), leukocyte-associated immunoglobulin like receptor 1 (LAIR1), CD244, lymphocyte activation gene 3 (LAG3), inducible T cell costimulator (ICOS), CD40 ligand gene (CD40LG), cytotoxic T lymphocyte antigen 4 (CTLA4), CD28, hepatitis A virus cellular receptor 2 (HAVCR2), CD80, programmed cell death 1 (PDCD1 or PD1), programmed cell death 1 ligand 2 (PDCD1LG2), CD27, TNF receptor superfamily member 25 (TNFRSF25), T cell immunoglobulin and ITIM domain (TIGIT), CD274 (PD-L1), and CD86 in ACC, LIHC, SKCM, and UVM. In contrast, FAM110A was negatively correlated with these genes in the BLCA, and LUSC (**Figure 5A**). Next, we verified the correlations between FAM110A and several immune checkpoint blocker genes, including PD1, PD-L1, CTLA-4, and LAG-3, in the TIMER 2.0 database, and the results were consistent with those of previous studies. The most significant positive correlation between FAM110A and these genes was observed in LIHC and SKCM, and the most significant negative correlation was observed in LGG and BLCA (**Figures 5B–E**; **Supplementary Table 1**).

The status of deficient mismatch repair (dMMR)/microsatellite instability-high (MSI-H) together with tumor mutational burden (TMB) are currently considered as promising predictive biomarkers for immunotherapy efficacy (14, 15). Significant correlations were found between FAM110A and several MMR-associated genes, such as MutL homolog 1 (MLH1), MutS homolog 2 (MSH2), and MutS homolog 6 (MSH6) in ACC, GBM, KIRC, LIHC, and STAD (**Figure 5F**). FAM110A expression was positively correlated with TMB in BRCA, LUAD, MESO, PAAD, STAD, and UCEC and negatively correlated with CDAD, DLBC, ESCA, and THYM (**Figure 5G**). Moreover, FAM110A expression was positively correlated with MSI in CESC, GBM, HNSC, KIRC, LUAD, LUSC, PRAD, and THCA and negatively correlated with MSI in COAD, READ, and SKCM (**Figure 5H**). However, according to a published result in the TISIDB database, no significant difference of FAM110A mRNA expression level was detected between immunotherapy responders and non-responders (**Supplementary Table 2**), which could be due to the small sample size in this study. The correlation between FAM110A expression and immunotherapy response still needs further in-depth study.

### 2.6 Correlation of FAM110A expression with immune infiltration

**Figure 6:**
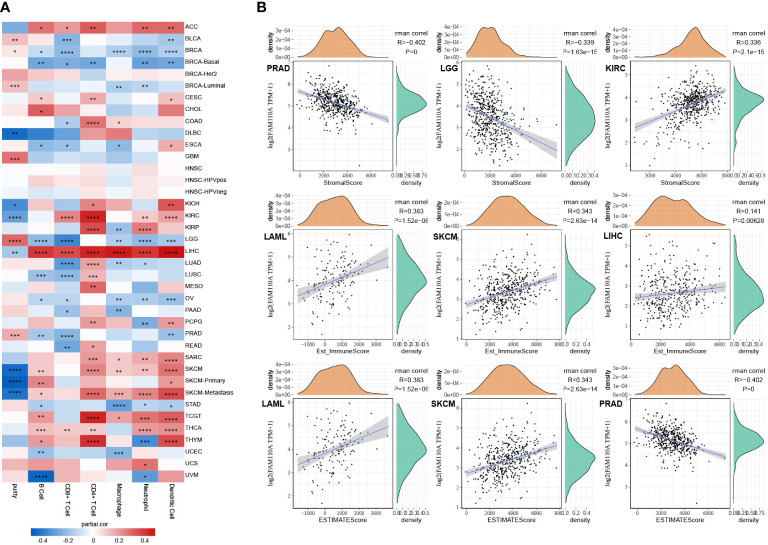
Correlation of FAM110A expression with immune infiltration. **(A)** Connection between FAM110A expression and the degree of immune cell infiltration in multiple malignancies using the infiltration scores of six immune cell types (B cell, CD4+ T cell, CD8+ T cell, neutrophil, macrophage, and dendritic cell) accessible in the TIMER database. (*P<0.05, **P<0.01, ***P<0.001, ****P<0.0001) **(B)** The top three tumors with the most significant correlation of FAM110A expression with stroma score, immune score and estimate score.

We used the TIMER database to explore the connection between FAM110A expression levels and the degree of tumor-infiltrating immune cell (TIIC) infiltration in pan-cancer (12). The correlation coefficients of purity and six TIICs (B cells, CD4+ T cells, CD8+ T cells, neutrophils, macrophages, and dendritic cells) collected from the TIMER database are shown in the form of heatmaps (**Figure 6A**). The most obvious positive correlation between immune cell infiltration and FAM110A was found in LIHC. In contrast, the strongest negative correlation between FAM110A expression and immune cell infiltration was observed in LGG. CD4+ cells exhibited the greatest significant coefficients among all cell types in multiple malignancies, including ACC, CESC, COAD, KIRC, KIRP, LIHC, LUAD, LUSC, MESO, TGCT, and THYM. A significant positive correlation between FAM110A expression and tumor purity was found in BRCA-luminal, GBM, and LGG, while a significant negative correlation was found between KIRC and SKCM.

We further investigated the link between FAM110A expression and tumor purity. We utilized the ESTIMATE algorithm to calculate the stroma score, immune score, and estimate score of relevant tumor samples based on the TCGA database and assessed the correlation between FAM110A expression levels and those scores. Based on our data, the three cancer types that showed the strongest association between FAM110A and the stroma score were PRAD, LGG, and KIRC. The three tumor types that showed the strongest association between FAM110A expression and immune score were LAML, SKCM, and LIHC. The three tumor types that showed the highest association between FAM110A and estimate scores were LAML, SKCM, and PRAD (**Figure 6B**). These results indicate that FAM110A expression was closely related to the degree of tumor purity and TIIC infiltration.

In addition, we assessed the effect of FAM110A on the infiltration levels of various immune cells in the TME, based on the expression of immune gene markers. Because of the contradictory roles played by FAM110A in prognosis and its association with immune checkpoints, we selected LIHC and BLCA as representative tumor types for subsequent analyses. PRAD served as a negative control because the expression of FAM110A did not seem to have a significant effect on PRAD prognosis. Results from the TIMER 2.0 database revealed a significant positive correlation between FAM110A expression and the expression of CD8+ T cells, T cells (general), monocytes, tumor-associated macrophages, M2 macrophages, dendritic cells, T helper type 1 (Th1), and exhausted T cells in LIHC. In BLCA, FAM110A and these gene markers were negatively correlated. As expected, no significant correlation was observed between FAM110A expression and TIIC markers in PRAD (**Table 1**). Analyses of the GEPIA database obtained similar results (**Supplementary Table 3**).

**Table 1:** Correlation analysis between FAM110A and related gene markers of immune cells in TIMER 2.0 (***P<0.001).

Description	Gene markers	LIHC(n=371)				BLCA(n=408)				PRAD(n=498)			
		None		Purity		None		Purity		None		Purity	
		rho	P	rho	P	rho	P	rho	P	rho	P	rho	P
CD8+Tcell	CD8A	0.391	***	0.369	***	-0.194	***	-0.152	0.003	-0.175	***	-0.066	0.180
	CD8B	0.399	***	0.367	***	-0.190	***	-0.151	0.004	0.039	0.383	0.115	0.019
T cell(general)	CD3D	0.537	***	0.535	***	-0.120	0.015	-0.044	0.401	-0.109	0.015	-0.014	0.774
	CD3E	0.442	***	0.442	***	-0.154	0.002	-0.086	0.098	-0.171	***	-0.073	0.137
	CD2	0.458	***	0.452	***	-0.148	0.003	-0.079	0.130	-0.152	***	-0.037	0.448
B cell	CD19	0.405	***	0.363	***	-0.115	0.020	-0.060	0.249	-0.068	0.129	0.004	0.931
	CD79A	0.387	***	0.359	***	-0.100	0.044	-0.034	0.517	-0.123	0.006	-0.050	0.308
Monocyte	CD86	0.526	***	0.526	***	-0.222	***	-0.189	***	-0.249	***	-0.140	0.004
	CD115 (CSF1R)	0.436	***	0.419	***	-0.205	***	-0.178	***	-0.242	***	-0.143	0.003
TAM	CCL2	0.415	***	0.383	***	-0.195	***	-0.154	0.003	-0.056	0.213	0.028	0.566
	CD68	0.327	***	0.291	***	-0.165	***	-0.144	0.006	-0.222	***	-0.142	0.004
	IL10	0.410	***	0.379	***	-0.210	***	-0.189	***	-0.207	***	-0.098	0.046
M1 Macrophage	INOS (NOS2)	0.051	0.325	0.037	0.492	0.010	0.840	0.059	0.261	-0.015	0.740	0.067	0.174
	IRF5	0.398	***	0.392	***	0.271	***	0.283	***	0.015	0.732	0.036	0.468
M2 Macrophage	CD163	0.235	***	0.190	***	-0.248	***	-0.230	***	-0.286	***	-0.202	***
	VSIG4	0.310	***	0.268	***	-0.240	***	-0.209	***	-0.258	***	-0.167	***
	MS4A4A	0.269	***	0.231	***	-0.272	***	-0.266	***	-0.286	***	-0.201	***
Neutrophils	CD66b(CEACAMB)	0.071	0.170	0.069	0.202	-0.031	0.528	-0.047	0.364	0.013	0.774	0.033	0.508
	CD11b (ITGAM)	0.461	***	0.441	***	-0.173	***	-0.147	0.005	-0.208	***	-0.108	0.027
	CCR7	0.276	***	0.245	***	0.033	0.508	0.058	0.267	-0.127	0.005	-0.026	0.603
NK cell	KIR2DL1	0.051	0.327	-0.006	0.918	-0.098	0.047	-0.058	0.270	-0.013	0.779	0.042	0.395
	KIR2DL3	0.222	***	0.213	***	-0.110	0.027	-0.074	0.157	-0.006	0.898	-0.008	0.873
	KIR2DL4	0.275	***	0.241	***	-0.110	0.026	-0.069	0.189	0.076	0.091	0.131	0.008
	KIR3DL1	0.054	0.300	0.024	0.664	-0.074	0.137	-0.037	0.475	-0.088	0.049	-0.071	0.151
	KIR3DL2	0.151	0.004	0.123	0.023	-0.093	0.060	-0.053	0.313	0.013	0.772	0.041	0.403
	KIR3DL3	0.084	0.107	0.072	0.179	0.030	0.547	0.058	0.271	-0.082	0.067	-0.135	0.006
	KIR2DS4	0.118	0.023	0.094	0.082	-0.080	0.105	-0.018	0.735	-0.054	0.232	-0.046	0.353
Dendritic cell	HLA-DPB1	0.428	***	0.388	***	-0.140	0.005	-0.096	0.066	-0.063	0.157	0.037	0.452
	HLA-DQB1	0.380	***	0.335	***	-0.095	0.055	-0.032	0.538	-0.122	0.007	-0.055	0.264
	HLA-DRA	0.381	***	0.339	***	-0.107	0.031	-0.060	0.247	-0.209	***	-0.102	0.037
	HLA-DPA1	0.366	***	0.329	***	-0.131	0.008	-0.088	0.091	-0.204	***	-0.092	0.062
	BCDA-1 (CD1C)	0.306	***	0.266	***	-0.103	0.037	-0.052	0.316	-0.135	0.003	-0.008	0.873
	BDCA-4 (NRP1)	0.231	***	0.198	***	-0.331	***	-0.315	***	-0.045	0.313	-0.005	0.912
	CD11c (ITGAX)	0.529	***	0.526	***	-0.204	***	-0.172	***	-0.101	0.025	-0.029	0.551
Th1	TBX21	0.299	***	0.263	***	-0.162	0.001	-0.108	0.038	-0.083	0.064	-0.006	0.908
	STAT4	0.356	***	0.344	***	-0.202	***	-0.143	0.006	-0.141	0.002	-0.037	0.449
	STAT1	0.358	***	0.345	***	-0.070	0.160	-0.017	0.749	-0.175	***	-0.068	0.169
	IFN-g (IFNG)	0.378	***	0.345	***	-0.100	0.043	-0.056	0.285	-0.109	0.015	-0.023	0.642
	TNF-a(TNF)	0.467	***	0.454	***	-0.031	0.527	0.014	0.788	-0.096	0.033	0.022	0.652
Th2	GATA3	0.464	***	0.456	***	0.347	***	0.344	***	0.004	0.924	0.132	0.007
	STAT6	0.005	0.921	0.000	0.997	0.238	***	0.248	***	-0.150	***	-0.099	0.043
	STAT5A	0.421	***	0.385	***	-0.022	0.655	0.016	0.758	-0.138	0.002	-0.027	0.588
	IL13	0.196	***	0.176	0.001	-0.127	0.010	-0.087	0.097	-0.016	0.724	-0.022	0.660
Tfh	BCL6	0.046	0.380	0.058	0.286	0.239	***	0.235	***	-0.222	***	-0.180	***
	IL21	0.093	0.072	0.093	0.084	-0.111	0.024	-0.083	0.113	-0.057	0.206	-0.023	0.646
Th17	STAT3	0.149	0.004	0.121	0.024	0.000	0.994	0.037	0.474	-0.131	0.004	-0.032	0.512
	IL17A	0.009	0.867	0.026	0.625	0.136	0.006	0.159	0.002	-0.114	0.011	-0.036	0.470
Treg	FOXP3	0.268	***	0.279	***	-0.148	0.003	-0.083	0.111	-0.081	0.073	-0.035	0.480
	CCR8	0.410	***	0.405	***	-0.164	***	-0.112	0.032	-0.163	***	-0.081	0.097
	STAT5B	0.090	0.083	0.120	0.025	-0.004	0.935	-0.014	0.795	-0.249	***	-0.148	0.002
	TGFb (TGFB1)	0.481	***	0.472		-0.132	0.007	-0.119	0.022	-0.073	0.104	-0.011	0.824
Tex	PD-1 (PDCD1)	0.495	***	0.487	***	-0.166	***	-0.113	0.030	-0.022	0.624	0.037	0.448
	CTLA4	0.518	***	0.514	***	-0.164	***	-0.097	0.062	-0.049	0.272	0.032	0.513
	LAG3	0.426	***	0.404	***	-0.181	***	-0.131	0.012	-0.033	0.465	0.031	0.523
	TIM-3 (HAVCR2)	0.538	***	0.546	***	-0.219	***	-0.196	***	-0.211	***	-0.107	0.029
	GZMB	0.294	***	0.250	***	-0.181	***	-0.117	0.025	-0.031	0.492	0.060	0.219

### 2.7 FAM110A-related genes are closely correlated with immue response in LIHC

**Figure 7:**
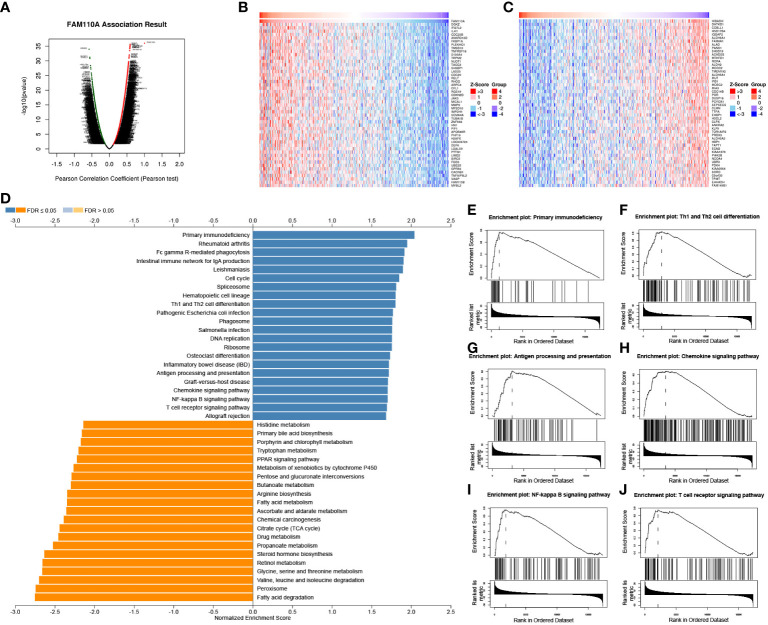
GSEA of FAM110A in the TCGA LIHC cohort. **(A)** Correlations between FAM110A and genes differentially expressed in LIHC. **(B, C)** Heat maps of the most 50 significant genes positively and negatively correlated with FAM110A in LIHC. **(D-J)** KEGG enrichment analyses showed that primary immunodeficiency, Th1 and Th2 cell differentiation, antigen processing and presentation, chemokine signaling pathway, NF-kappa B signaling pathway, and T cell receptor signaling pathway were enriched.

Our previous results revealed that FAM110A is closely related to patient prognosis and immunity in pan-cancer. Since the strongest correlation between FAM110A expression and immune infiltration was observed in LIHC, we used LIHC as an example to verify the potential function of FAM110A using the LinkedOmics database. We analyzed the co-expression of genes associated with FAM110A in LIHC (**Figure 7A**), and the top 50 genes with the most significant positive or negative correlations with FAM110A are displayed using a heat map (**Figure 7B, C**).

The Gene Set Enrichment Analysis (GSEA) analysis based on FAM110A-related genes in LIHC showed that GO biological process terms were mainly enriched for interferon-gamma production, interleukin-4 production, interleukin-10 production, T cell activation, B cell activation, myeloid dendritic cell activation, and adaptive immune response (**Supplementary Figure 2**). Kyoto Encyclopedia of Genes and Genomes (KEGG) pathway enrichment analysis showed that the major enriched pathways were primary immunodeficiency, Th1 and Th2 cell differentiation, antigen processing and presentation, chemokine signaling, NF-kappa B signaling, and T cell receptor signaling (**Figure 7D-J**).

### 2.8 FAM110A is associated with poor prognosis, immune infiltration, and immune checkpoints in LIHC

**Figure 8:**
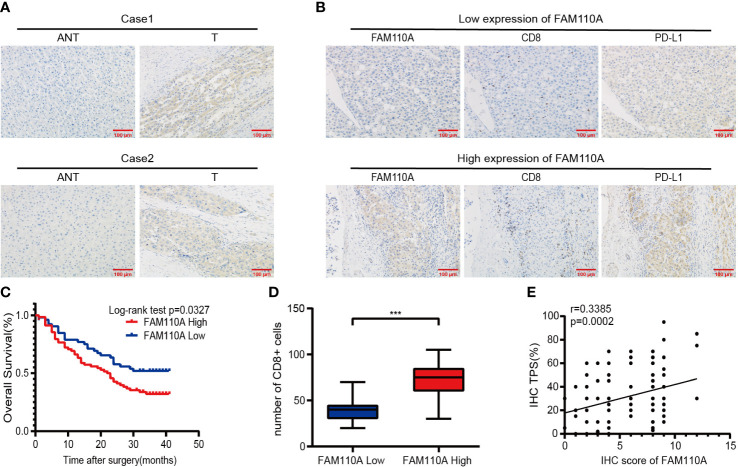
FAM110A expression correlated with immune infiltration and poor prognosis in LIHC. **(A)** Representative FAM110A staining image in cancerous and adjacent normal tissues. **(B)** Representative IHC staining images of LIHC tissues with FAM110A-high and low expression. Positive CD8, PD-L1 expression cells were shown. Scale bars, 100 μm. **(C)** Kaplan–Meier survival curves for OS of patients with LIHC based on the expression status of FAM110A.*p < 0.05. **(D)**The number of CD8+ T cells in LIHC tissues with high or low FAM110A expression.***P<0.001. **(E)** The correlation of FAM110A with PD-L1 protein expression was evaluated by Pearson’s correlation.

To further verify the expression of FAM110A in LIHC, IHC analysis was performed to detect the expression level of FAM110A protein in 120 randomly selected tumor tissues and paired adjacent non-tumor tissues from patients with LIHC. Our results revealed that FAM110A protein expression was significantly increased in tumor tissues compared to that in matched non-tumor adjacent tissues, the subcellular localization of FAM110A was in the nucleoplasm and cytoplasm (**Figure 8A**). According to the IHC scoring criteria, the high expression rate of FAM110A in tumor tissues was 56.7% (68/120) and the low expression rate was 43.3% (52/120).

To verify the effect of FAM110A on poor prognosis in patients with LIHC, all randomly selected patients were divided into high and low FAM110A expression groups (**Figure 8B**), and the clinical follow-up data of those patients were analyzed through Kaplan-Meier survival analysis and log-rank test. Our results showed that patients with high FAM110A expression were associated with worse prognosis than those with low FAM110A expression (**Figure 8C**).

Next, the correlation between FAM110A and immune infiltration and immune checkpoint genes was verified. We evaluated the degree of immune infiltration and PD-L1 expression in serial sections of the specimens from the same patient. The number of CD8+ lymphocytes infiltrating the tissues of the patients was significantly higher than that of the patients with low FAM110A expression (**Figure 8B, D**), and the PD-L1 protein expression levels were positively correlated with the FAM110A expression levels (**Figure 8B, E**).

## 3 Discussion

FAM110A is a centrosome-associated protein localized at the mitotic spindle and spindle poles during mitosis (7). Perez et al. revealed that aberrant expression of FAM110A may result in dysregulation of the cell cycle (12), which is regarded as a typical characteristic of cancer cells (16). Huang et al. demonstrated that FAM110A is an oncogene that promotes the malignant behavior of cancer cells and tumorigenesis in pancreatic cancer (8). In this study, we conducted a comprehensive bioinformatics analysis of FAM110A using multiple public databases.

Our results showed that FAM110A mRNA is widely distributed and overexpressed in most cancer tissues compared to that in normal tissues. Moreover, the expression level of FAM110A mRNA correlated with the clinicopathological stages of BRCA, LIHC, SKCM, and THCA. Our findings also demonstrated a significant correlation between FAM110A mRNA expression and CNV and methylation in pan-cancers. In view of the abnormal expression levels of FAM110A in tumors, the Kaplan–Meier method and COX regression analysis were conducted, and the results demonstrated that FAM110A may serve as a potential prognostic biomarker for a variety of cancers, especially LIHC. FAM110A is closely related to multiple immune checkpoint genes, and its expression levels may indirectly reflect the abundance of these two immune infiltrates in the TME. As a result, we propose that FAM110A plays a delicate role in tumor initiation or development based on differential expression profiles and may affect immunotherapy efficacy to some extent.

Tumor immunotherapy aims to boost the natural immune system and relies on the patients’ own immune function to eliminate cancer cells and tumor tissues (17, 18). Through the successful application of monoclonal antibodies, cytokines, cellular immunotherapy, and vaccines, immunotherapy has revolutionized cancer treatment (19). Immune-related gene expression is regarded as a predictive marker for immunotherapy in a variety of cancers (20–22). Here, we analyzed the association of FAM110A with more than 40 immune checkpoint genes in pan-cancer based on the TCGA database and verified the correlation of FAM110A between several immune checkpoint genes, including PD-1, PD-L1, LAG-3, and CTLA-4 in the TIMER 2.0 database. Our data suggested that FAM110A had the strongest positive correlation with these immune checkpoint genes in cancers where FAM110A is considered an important risk factor, such as LIHC and SKCM. Meanwhile, in BLCA and LGG, where FAM110A served as a protective factor, the expression of FAM110A showed the strongest negative correlation with these immune inhibitor checkpoint genes. This may explain the association between FAM110A overexpression and poor prognosis in patients with cancer.

DNA mismatch repair (MMR) is an important DNA repair pathway that plays critical roles in DNA replication fidelity, mutation avoidance, and genome stability. MMR-deficiency leads to a hypermutated phenotype in the genome, which in turn leads to MSI (23). Specifically, MMR-deficient cancers tend to be more sensitive to immune checkpoint blockade (24). We found statistically significant correlations between FAM110A and MSI in several cancers; however, the correlation was not very strong (correlation coefficient<0.6). The association of FAM110A with cancer patient prognosis, especially immunotherapy efficacy, requires further clinical validation.

The immune TME, majorly represented by the TIICs, plays an important role in cancer therapeutics and patient prognosis (20, 25). In fact, a high density of TIICs within the TME is associated with better outcomes in several types of cancers (26–28). A thorough understanding of the factors involved in regulating immune infiltrates will aid in improving response rates and developing new therapeutic strategies (29). Results from the TIMER database showed that infiltration abundance of several TIICs, such as B cells, CD4+ T cells, CD8+ T cells, neutrophils, macrophages, and dendritic cells, were significantly correlated with the expression of FAM110A in multiple malignancies, especially in LIHC. Taking these findings together, we speculated that FAM110A is also significantly associated with immune-related functions and pathways in LIHC. We performed GO and KEGG enrichment analysis of FAM110A-related genes in LIHC and found highly significant enrichment of GO terms associated with immune function, including interferon-γ production, T cell activation, B cell activation, adaptive immune response, mast cell-mediated immunity, and positive regulation of cell activation. We also identified an enrichment of immune-related signaling pathways, including primary immunodeficiency, Th1 and Th2 cell differentiation, antigen processing and presentation, chemokine signaling, NF-kappa B signaling and T cell receptor signaling pathways, through KEGG pathway analysis. Based on our identification, FAM110A is involved in the activation of T cells as well as related immune pathways, which suggests that FAM110A plays an important role in the immune process. These results may explain the possible mechanism by which FAM110A promotes immune infiltration, and provids corroborating support for the role of FAM110A as an immunological biomarker.

Importantly, we further confirmed the abnormal expression of FAM110A in LIHC and the correlation between the expression of immune checkpoint PD-L1 protein and the immune infiltration degree of CD8+ cells by IHC experiments. The Kaplan-Meier plot and log-rank test demonstrated that high FAM110A expression leads to a worse prognosis in patients with LIHC. In addition, previous studies have shown that aberrant expression of FAM110A is associated with cell cycle dysregulation (12), which is considered to be a fundamental mechanism underlying malignant progression (30). And this fact may represent an important cause for the prognostic impact of FAM110A.

Due to the potential prognostic value of FAM110A, the expression level of FAM110A in postoperative tissue specimens can be used as one of the bases for assessing the prognosis of patients in various tumors, particularly in LIHC. Moreover, our fingdings also sets a new path in the field of tumor immunology. Based on the findings, more studies are expected to reveal the underlying mechanism of FAM110A regulating tumor immune microenvironment in the future, which would be beneficial for progresses of cancer immunotherapy.

There are still many limitations in this study. To begin with, some of our results are limited to a single approach or database, lacking mutual validation of data from multiple sources. Moreover, our bioinformatic results show that FAM110A is associated with poor prognosis of liver cancer and immune response. however, we are still uncertain whether FAM110A affects prognosis by regulating immune processes. In addition, although these findings have pointed to new directions for subsequent studies, the potential biological function process and molecular mechanism involved still deserve detailed experimental validation.

In general, we performed a comprehensive analysis of FAM110A using bioinformatics methods, revealing the important role of FAM110A in prognosis and immune infiltration in multiple cancers, especially in LIHC. More importantly, our study provides a promising candidate for therapeutic targets and a new direction for future research.

## 4 Materials and methods

### 4.1 FAM110A expression analysis

The HPA (https://www.proteinatlas.org) database was used to explore the mRNA and protein expression levels of FAM110A in normal human tissues. The expression level of FAM110A gene in a variety of cancer tissues was obtained through the “Gene_DE” module in the TIMER 2.0 (http://timer.cistrome.org/) (31). The RNA-seq data of normal and tumor samples were collected from the TCGA (http://cancergenome.nih.gov) and GTEx (http://commonfund.nih.gov/GTEx/) projects. We used the “Stage plot” function in the Gene Expression Profiling Interactive Analysis (GEPIA; http://gepia.cancer-pku.cn/) (32) database to analyze the correlation between FAM110A expression and tumor stage. The TISIDB (http://cis.hku.hk/TISIDB/) database (33) was used to detect difference of FAM110A mRNA expression level between immunotherapy responders and non-responders.

### 4.2 CNV and methylation analysis

The Gene Set Cancer Analysis (GSCA; http://bioinfo.life.hust.edu.cn/GSCA/#/) database is a powerful bioinformatics analysis tool which mainly integrates the mRNA expression, mutation, immune infiltrates, methylation data from the TCGA database (34), The “mutation” module in the GSCA database was used to analyze CNVs and methylation of FAM110A as well as their correlation with mRNA expression levels. SangerBox (http://vip.sangerbox.com/) is a comprehensive, user-friendly bioinformatics analysis platform (35). The relationship between FAM110A and methyltransferase genes expression was investigated by Sangerbox platform.

### 4.3 Survival analysis

We verified the prognostic value of FAM110A based on clinical data from the TCGA database, Xiantao Academic Online Website (https://www.xiantao.love/) was used for bioinformatics analysis based on the R language. In the R environment, RNA sequencing data in fragments per kilobase per million format were transformed into transcripts per million reads format. The “Survival” (version 3.2-10) and “survminer” (version 0.4.9) packages were used for statistical analysis and visualization, respectively. The statistical significance of OS and DSS between the high and low FAM110A expression groups in patients with 33 cancer types was analyzed by univariate Cox regression. Statistical significance was set at P < 0.05.

### 4.4 Immune infiltration analysis

The correlation data between FAM110A expression and six types of TIICs (B cell, CD4+ T cell, CD8+ T cell, neutrophil, macrophage, and dendritic cell) were obtained from the “GENE” module in the TIMER (https://cistrome.shinyapps.io/timer/) database (36). Estimation of Stromal and Immune cells in Malignant Tumor tissues using Expression data (ESTIMATE) is a method that uses gene expression signatures to infer the proportion of mesenchymal and immune cells in tumor samples. We use the “ESTIMATE” package to calculate the immune score, stromal score and estimate score of relate samples respectively. The correlation between those scores and the expression of FAM110A was explored through SangerBox platform.

### 4.5 Co-expressed genes and gene enrichment analysis

The LinkedOmics (http://www.linkedomics.org/login.php) database (37) is a multi-omics database that integrates multi-omics data and clinical data for 32 cancer types and 11,158 patients from the TCGA project. We selected the data set “LIHC cohort”, data type “RNAseq”, and the statistical method “Pearson correlation test” to analyze the co-expression genes of FAM110A in LIHC. The “Gene Set Enrichment Analysis (GSEA)” tool was then used to conduct the GO_BP term search and KEGG pathway enrichment analysis to those FAM110A-related genes.

### 4.6 Patients and tissue specimens

All clinical samples were obtained from the First Affiliated Hospital of Nanchang University, China. Formalin-fixed, paraffin-embedded samples from 120 patients were collected from January 2019 to December 2019. All samples were collected with the consent of the patients and the study was approved by the Ethics Committee of the First Affiliated Hospital of Nanchang University. All patient specimens and clinical data used in this study complied with the principles of the Declaration of Helsinki.

### 4.7 Immunohistochemistry analysis

Paraffin-embedded tissue sections were degreased by immersion in xylene for 10 min and hydrated in various concentrations of alcohol, followed by antigen retrieval using ethylenediaminetetraacetic acid solution, boiled in a pressure cooker for 1.5 min, and cooled down to room temperature naturally. The slides were then immersed in 3% H_2_O_2_ for 10 min to eliminate endogenous peroxidase activity. After washing with phosphate-buffered saline (PBS), the sections were incubated with the FAM110A antibody (1:20, sc-376464, SANTA CRUZ), anti-CD274 antibody (1:200, 66248-1-Ig, Proteintech), or CD8 antibody (1:200, 85336S, Cell Signaling) overnight at 4°C. After three times of washing with PBS, the sections were incubated with secondary antibody for 20 min at 37°C and stained using diaminobenzidine solution. IHC scores were calculated according to the staining intensity and the corresponding percentage of positive cells, tumor proportion score (TPS) were calculated according to the percentage of tumor cells showing partial or complete cell membrane staining of PD-L1. Two blinded, independent pathologists observed the results under a light microscope.

### 4.8 Statistical analysis

For bioinformatic data, the whole dataset was filtered by deleting missing and duplicated data, and all statistical analyses and visualizations were conducted using the R software (version 3.6.3) (http://www.rproject.org/). The correlation between FAM110A and immune checkpoint and MMR genes was evaluated using Pearson’s correlation test. The Wilcoxon rank-sum test was used for differential expression analysis of FAM110A between cancer and normal tissues, and the results were visualized using the “ggplot2” package (version 3.3.3). For clinical data, we compared the two groups using a t-test for continuous variables. GraphPad Prism 8 was used for statistical analysis and visualization and p < 0.05 was considered statistically significant.

## Data availability statement

The original contributions presented in the study are included in the article/**Supplementary Material**. Further inquiries can be directed to the corresponding authors.

## Ethics statement

The studies involving human participants were reviewed and approved by Ethics Committee of the First Affiliated Hospital of Nanchang University. Written informed consent for participation was not required for this study in accordance with the national legislation and the institutional requirements.

## Author contributions

JD, WL, and LL designed the study and guided work. HZ, and QS wrote the manuscript. JC, and QW conducted data collection and analyses. XL assisted in the collection of tissue samples. RR performed immunohistochemistry. SZ, and XD collected clinical information. JX helped the revision. All authors contributed to the article and approved the submitted version.
