# Supplementary figures and images for: Pan-cancer analysis reveals potential of FAM110A as a prognostic and immunological biomarker in human cancer
Source: Front Immunol. 2023 Feb 27;14:1058627. doi: 10.3389/fimmu.2023.1058627 (PMC10008925; doi:10.3389/fimmu.2023.1058627)

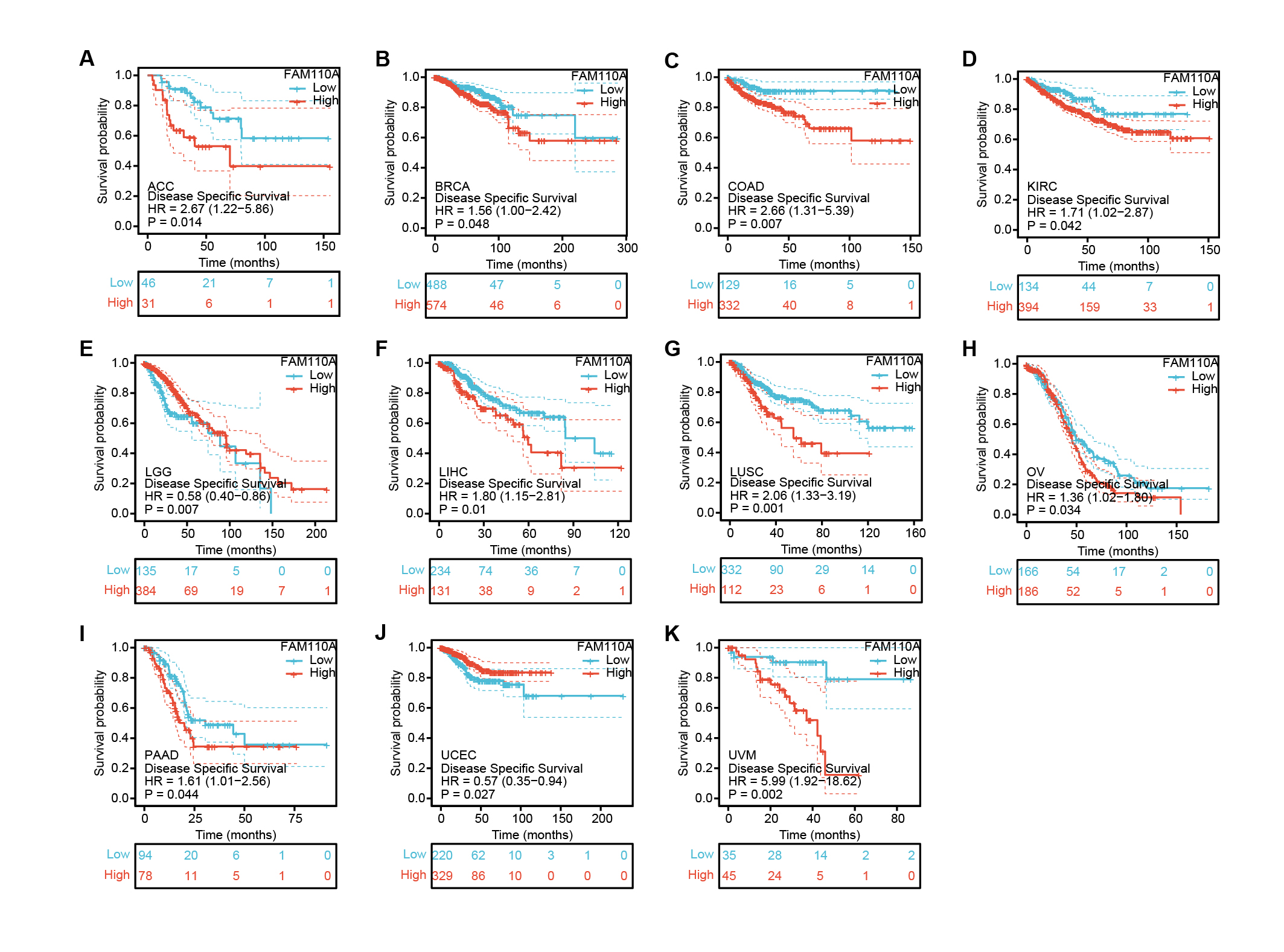

Supplement: Supplementary file 1 [file Image_1.jpeg]

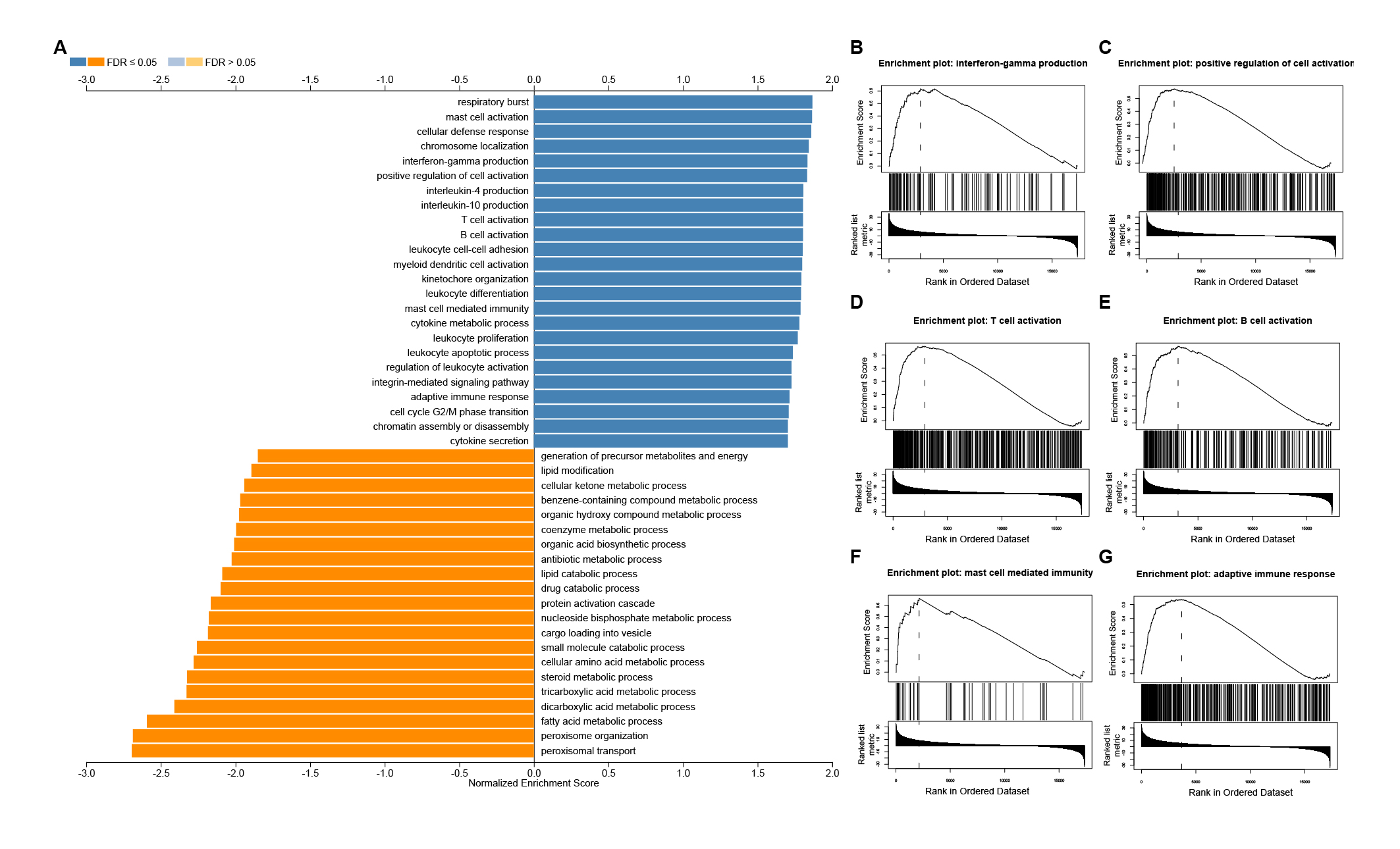

Supplement: Supplementary file 2 [file Image_2.jpeg]
